# Catheter ablation of atrial fibrillation in patients with cardiac amyloidosis and sarcoidosis: procedural findings and outcomes

**DOI:** 10.1093/europace/euaf100

**Published:** 2025-06-20

**Authors:** Alireza Oraii, Balaram Krishna J Hanumanthu, Adrian Petzl, Ting-Wei Ernie Liao, Arian Afzalian, Oriol Rodriguez-Queralto, Corentin Chaumont, Jenna Spears, Timothy M Markman, Matthew C Hyman, Cory M Tschabrunn, Gustavo Guandalini, Andres Enriquez, Poojita Shivamurthy, Ramanan Kumareswaran, Rajat Deo, Victor A Ferrari, Michael P Riley, David Lin, David J Callans, Gregory E Supple, Robert D Schaller, David S Frankel, Fermin C Garcia, Saman Nazarian, Sanjay Dixit, Francis E Marchlinski

**Affiliations:** Section of Cardiac Electrophysiology, Division of Cardiovascular Medicine, Department of Medicine, Hospital of the University of Pennsylvania, 1 Convention Ave, Pavilion - 2nd floor City Side, Philadelphia, PA 19104, USA; Section of Cardiac Electrophysiology, Division of Cardiovascular Medicine, Department of Medicine, Hospital of the University of Pennsylvania, 1 Convention Ave, Pavilion - 2nd floor City Side, Philadelphia, PA 19104, USA; Section of Cardiac Electrophysiology, Division of Cardiovascular Medicine, Department of Medicine, Hospital of the University of Pennsylvania, 1 Convention Ave, Pavilion - 2nd floor City Side, Philadelphia, PA 19104, USA; Section of Cardiac Electrophysiology, Division of Cardiovascular Medicine, Department of Medicine, Hospital of the University of Pennsylvania, 1 Convention Ave, Pavilion - 2nd floor City Side, Philadelphia, PA 19104, USA; Section of Cardiac Electrophysiology, Division of Cardiovascular Medicine, Department of Medicine, Hospital of the University of Pennsylvania, 1 Convention Ave, Pavilion - 2nd floor City Side, Philadelphia, PA 19104, USA; Section of Cardiac Electrophysiology, Division of Cardiovascular Medicine, Department of Medicine, Hospital of the University of Pennsylvania, 1 Convention Ave, Pavilion - 2nd floor City Side, Philadelphia, PA 19104, USA; Section of Cardiac Electrophysiology, Division of Cardiovascular Medicine, Department of Medicine, Hospital of the University of Pennsylvania, 1 Convention Ave, Pavilion - 2nd floor City Side, Philadelphia, PA 19104, USA; Section of Cardiac Electrophysiology, Division of Cardiovascular Medicine, Department of Medicine, Hospital of the University of Pennsylvania, 1 Convention Ave, Pavilion - 2nd floor City Side, Philadelphia, PA 19104, USA; Section of Cardiac Electrophysiology, Division of Cardiovascular Medicine, Department of Medicine, Hospital of the University of Pennsylvania, 1 Convention Ave, Pavilion - 2nd floor City Side, Philadelphia, PA 19104, USA; Section of Cardiac Electrophysiology, Division of Cardiovascular Medicine, Department of Medicine, Hospital of the University of Pennsylvania, 1 Convention Ave, Pavilion - 2nd floor City Side, Philadelphia, PA 19104, USA; Section of Cardiac Electrophysiology, Division of Cardiovascular Medicine, Department of Medicine, Hospital of the University of Pennsylvania, 1 Convention Ave, Pavilion - 2nd floor City Side, Philadelphia, PA 19104, USA; Section of Cardiac Electrophysiology, Division of Cardiovascular Medicine, Department of Medicine, Hospital of the University of Pennsylvania, 1 Convention Ave, Pavilion - 2nd floor City Side, Philadelphia, PA 19104, USA; Section of Cardiac Electrophysiology, Division of Cardiovascular Medicine, Department of Medicine, Hospital of the University of Pennsylvania, 1 Convention Ave, Pavilion - 2nd floor City Side, Philadelphia, PA 19104, USA; Section of Cardiac Electrophysiology, Division of Cardiovascular Medicine, Department of Medicine, Hospital of the University of Pennsylvania, 1 Convention Ave, Pavilion - 2nd floor City Side, Philadelphia, PA 19104, USA; Section of Cardiac Electrophysiology, Division of Cardiovascular Medicine, Department of Medicine, Hospital of the University of Pennsylvania, 1 Convention Ave, Pavilion - 2nd floor City Side, Philadelphia, PA 19104, USA; Section of Cardiac Electrophysiology, Division of Cardiovascular Medicine, Department of Medicine, Hospital of the University of Pennsylvania, 1 Convention Ave, Pavilion - 2nd floor City Side, Philadelphia, PA 19104, USA; Section of Cardiac Electrophysiology, Division of Cardiovascular Medicine, Department of Medicine, Hospital of the University of Pennsylvania, 1 Convention Ave, Pavilion - 2nd floor City Side, Philadelphia, PA 19104, USA; Section of Cardiac Electrophysiology, Division of Cardiovascular Medicine, Department of Medicine, Hospital of the University of Pennsylvania, 1 Convention Ave, Pavilion - 2nd floor City Side, Philadelphia, PA 19104, USA; Section of Cardiac Electrophysiology, Division of Cardiovascular Medicine, Department of Medicine, Hospital of the University of Pennsylvania, 1 Convention Ave, Pavilion - 2nd floor City Side, Philadelphia, PA 19104, USA; Section of Cardiac Electrophysiology, Division of Cardiovascular Medicine, Department of Medicine, Hospital of the University of Pennsylvania, 1 Convention Ave, Pavilion - 2nd floor City Side, Philadelphia, PA 19104, USA; Section of Cardiac Electrophysiology, Division of Cardiovascular Medicine, Department of Medicine, Hospital of the University of Pennsylvania, 1 Convention Ave, Pavilion - 2nd floor City Side, Philadelphia, PA 19104, USA; Section of Cardiac Electrophysiology, Division of Cardiovascular Medicine, Department of Medicine, Hospital of the University of Pennsylvania, 1 Convention Ave, Pavilion - 2nd floor City Side, Philadelphia, PA 19104, USA; Section of Cardiac Electrophysiology, Division of Cardiovascular Medicine, Department of Medicine, Hospital of the University of Pennsylvania, 1 Convention Ave, Pavilion - 2nd floor City Side, Philadelphia, PA 19104, USA; Section of Cardiac Electrophysiology, Division of Cardiovascular Medicine, Department of Medicine, Hospital of the University of Pennsylvania, 1 Convention Ave, Pavilion - 2nd floor City Side, Philadelphia, PA 19104, USA; Section of Cardiac Electrophysiology, Division of Cardiovascular Medicine, Department of Medicine, Hospital of the University of Pennsylvania, 1 Convention Ave, Pavilion - 2nd floor City Side, Philadelphia, PA 19104, USA; Section of Cardiac Electrophysiology, Division of Cardiovascular Medicine, Department of Medicine, Hospital of the University of Pennsylvania, 1 Convention Ave, Pavilion - 2nd floor City Side, Philadelphia, PA 19104, USA; Section of Cardiac Electrophysiology, Division of Cardiovascular Medicine, Department of Medicine, Hospital of the University of Pennsylvania, 1 Convention Ave, Pavilion - 2nd floor City Side, Philadelphia, PA 19104, USA

**Keywords:** Atrial fibrillation, Catheter ablation, Sarcoidosis, Amyloidosis, Cardiomyopathies, Infiltrative

## Abstract

**Aims:**

The diagnosis of infiltrative cardiomyopathies has increased over last years. Catheter ablation is becoming the preferred approach for managing atrial fibrillation (AF) in these patients. This study aims to characterize differences in procedural findings during AF ablation in patients with and without infiltrative cardiomyopathies.

**Methods and results:**

Patients with cardiac amyloidosis and cardiac sarcoidosis undergoing first-time AF ablation were propensity score matched in 1:4 ratio to separate reference groups that received trigger provocative manoeuvres (isoproterenol infusion and/or atrial burst pacing) and had no prior cardiac surgery. Non-pulmonary vein (PV) triggers [defined as ectopic foci initiating AF or sustained focal atrial tachycardia (AT)] and macro-reentrant atrial flutters (AFLs) were then mapped and targeted. Recurrence was defined as AF/AT/AFL ≥ 30 s after 90-day blanking period. Twenty-four patients with cardiac amyloidosis were matched to 96 controls, and 17 patients with cardiac sarcoidosis were matched to 68 controls. Non-PV triggers were more frequent in patients with cardiac amyloidosis {29.2% vs. 8.3%; odds ratio [OR] = 4.5 [95% confidence interval (CI): 1.4–14.2]} and cardiac sarcoidosis [17.6% vs. 7.4%; OR = 2.7 (95% CI: 0.6–12.6)] compared with their reference groups. Patients with cardiac amyloidosis also had a higher incidence of left atrial macro-reentrant flutters [37.5% vs. 6.3%; OR = 9.0, (95% CI: 2.8–29.0)]. One-year recurrence rate was similar between patients with cardiac sarcoidosis and controls (33.3% vs. 33.9%; *P* = 0.965) but higher in patients with cardiac amyloidosis vs. controls (47.4% vs. 27.1%; *P* = 0.049).

**Conclusion:**

Patients with infiltrative cardiomyopathies exhibit higher rates of non-PV triggers and left AFLs during first-time AF ablation. Those with cardiac amyloidosis experience higher arrhythmia recurrence rates compared with controls.

## Graphical Abstract

**Graphical Abstract:**
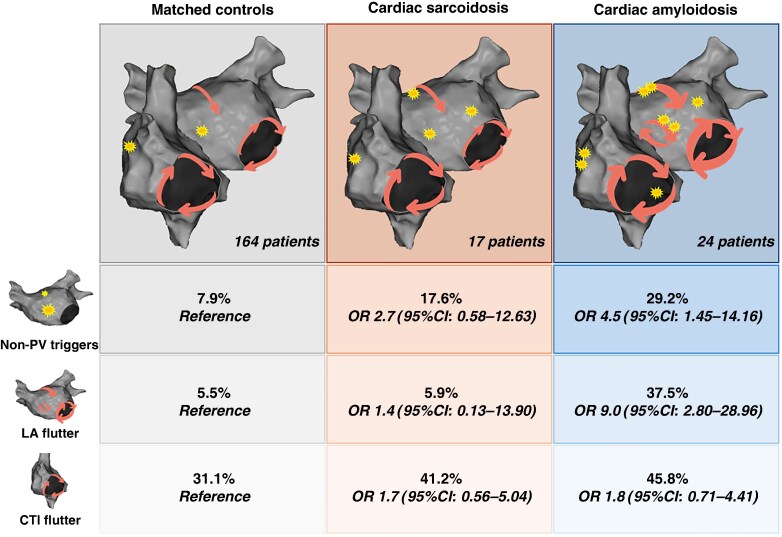

What’s new?

- Patients with infiltrative cardiomyopathies undergoing first-time atrial fibrillation ablation are more frequently found to have left atrial voltage abnormalities than matched reference groups.
- These patients have two- to four-fold higher incidence of focal non-pulmonary vein triggers initiating atrial fibrillation or sustained focal atrial tachycardia during first-time atrial fibrillation ablation.
- Patients with cardiac amyloidosis are also at particularly higher risk of having left atrial macro-reentrant flutters during first-time atrial fibrillation ablation.
- After targeting identified triggers and macro-reentrant flutters, patients with cardiac sarcoidosis have comparable outcomes with their matched reference group, but patients with cardiac amyloidosis still experience higher recurrence rates within 1 year after catheter ablation.

## Introduction

Infiltrative cardiomyopathies, particularly cardiac amyloidosis and sarcoidosis, have gained significant attention in recent years due to advancements in non-invasive diagnostic modalities and the development of disease-modifying therapies. These patients face a markedly increased risk of heart rhythm disorders, particularly atrial fibrillation (AF).^1,2^ The likelihood of developing AF in patients with infiltrative cardiomyopathies is up to 10 times higher than in the general population, with a prevalence of 15–30% in cardiac sarcoidosis and 50–70% in cardiac amyloidosis.^3–6^

Restoration and maintenance of sinus rhythm have been correlated with better survival in patients with AF and cardiac amyloidosis.^4^ This benefit was greatest in those undergoing catheter ablation compared with those treated with medical therapy alone.^7^ Additionally, analyses from national administrative databases have shown that AF ablation in patients with cardiac amyloidosis or cardiac sarcoidosis has safety outcomes and complication rates comparable with those observed in patients without infiltrative cardiomyopathies.^8,9^ However, it still remains unclear whether sites of arrhythmogenic triggers and ablation targets in patients with infiltrative cardiomyopathies differ from those in the general population. The majority of previous studies on catheter ablation in patients with infiltrative cardiomyopathies are limited by small sample sizes, absence of comparator groups, and inclusion of ablation procedures for various supraventricular tachycardias.^10–12^ Notably, AF ablations constituted a minority of the procedures in these studies. Furthermore, no study to date has compared procedural findings across different types of infiltrative cardiomyopathies.

This study aimed to compare identified triggers and procedural findings during AF ablation between patients with and without infiltrative cardiomyopathies and to evaluate whether these findings differ based on the type of infiltrative cardiomyopathy. Additionally, we aimed to assess the comparative efficacy of AF ablation on arrhythmia-free survival in patients with and without infiltrative cardiomyopathy.

## Methods

### Study design and setting

The AF ablation registry at the Hospital of the University of Pennsylvania (HUP-AF registry) was used to identify all patients who underwent first-time AF ablation between January 2016 and December 2023. This registry prospectively collects detailed information regarding demographics, cardiovascular risk factors, comorbidities, echocardiographic data, and intraprocedural findings in patients undergoing AF ablation. All patients provided written informed consent to have their data prospectively collected. The study was approved by the institutional review board, and the research reported in this paper adhered to Helsinki Declaration guidelines.

### Study participants

We included patients diagnosed with cardiac amyloidosis or cardiac sarcoidosis. Cardiac amyloidosis was defined according to criteria outlined in the 2020 American Heart Association scientific statement for the diagnosis of cardiac amyloidosis.^13^ This included either histologic evidence on endomyocardial biopsy, Grade 2 or 3 cardiac uptake on technetium-99m pyrophosphate (PYP) scintigraphy scan, or presence of confirmed light-chain amyloidosis with imaging findings consistent with cardiac amyloidosis on echocardiogram and/or cardiac magnetic resonance imaging (MRI). Cardiac sarcoidosis was defined based on criteria outlined in the 2024 American Heart Association scientific statement for diagnosis of cardiac sarcoidosis.^14^ This included histologic evidence on endomyocardial biopsy, confirmed diagnosis of extracardiac sarcoidosis with clinical or imaging findings consistent with cardiac sarcoidosis on fluorodeoxyglucose (FDG) positron emission tomography (PET) scan and/or cardiac MRI, or presence of clinical and imaging findings consistent with cardiac sarcoidosis on both FDG-PET and cardiac MRI in the absence of extracardiac sarcoidosis. Alternative causes for the observed clinical and imaging findings were excluded.

Furthermore, we included a matched reference group for patients with cardiac amyloidosis and a separate matched reference group for patients with cardiac sarcoidosis to facilitate characterization of differences in procedural findings and ablation outcomes. Patients eligible to serve as reference groups were selected from the remaining study sample that received any trigger provocative manoeuvres (described below), lacked a history of heart/lung transplantation or a prior cardiac surgery, and did not have a diagnosis of infiltrative cardiomyopathy.

### Procedural details

Details of our institutional trigger provocation protocol, standard catheter positioning, and trigger localization have been previously described.^15–17^ In brief, a decapolar catheter is positioned inside the coronary sinus (CS) with the proximal electrode at the CS ostium and another decapolar catheter along the crista terminalis with the distal electrode extending into the superior vena cava to facilitate primary regionalization of triggers. After cardioversion of AF (if present at baseline), patients undergo 3D electroanatomical and voltage mapping with multipolar mapping catheters (PENTARAY/OCTARAY, Biosense Webster Inc.; Advisor HD Grid, Abbot Inc.) to define left atrial anatomy and areas of voltage abnormality. Coronary sinus pacing was performed to stabilize the catheters and facilitate voltage mapping. Low voltage areas were defined as areas ≥ 1cm^2^ with bipolar voltage of <0.5 mV during sinus/paced rhythm. Thereafter, wide circumferential antral pulmonary vein (PV) isolation (PVI) is performed with radiofrequency catheter ablation under general anaesthesia with jet ventilation. After confirmation of PVI with entrance and exit block, patients undergo standard trigger provocative manoeuvres, which consist of incremental isoproterenol infusion at 3, 6, 12, and up to 20–30 µg/min as haemodynamically tolerated and/or rapid atrial burst pacing with 15-beat drive trains starting at a cycle length of 250 ms followed by 10 ms decrements to 180 ms or loss of 1:1 capture in an attempt to induce atrial tachycardias (ATs), or induction of AF followed by cardioversion during residual isoproterenol infusion at a rate of 3–6 µg/min or during isoproterenol washout from maximum incremental dose. These pharmacologic challenges and pacing manoeuvres may be partially or entirely deferred at the operator's discretion, particularly if there is a perceived risk associated with haemodynamic intolerance or prolonged anaesthesia.

Non-PV triggers were defined as atrial premature depolarizations outside of PVs that initiated sustained or non-sustained AF or sustained focal AT. Non-sustained AT and isolated frequent non-PV atrial premature depolarizations that did not trigger AF were not considered non-PV triggers. Once the non-PV trigger was regionalized, the multipolar mapping and ablation catheters were manipulated to the suspected region of origin for more detailed mapping after repeated inductions and cardioversions (if necessary). The identified site of earliest activation was targeted with focal ablation to render the trigger non-inducible after repeating the provocative manoeuvres. Triggers originating from left atrial posterior wall or superior vena cava were targeted with posterior wall isolation or superior vena cava isolation, respectively. In patients where precise localization was challenging, the anatomic target (e.g. crista terminalis and Eustachian ridge) with the earliest activation was identified and ablated more extensively avoiding lesions where phrenic capture or proximity to the sinus node or atrioventricular conduction system was evident. Macro-reentrant atrial flutters (AFLs) were mapped using activation and entrainment mapping to identify the participating macro-reentry circuits [e.g. cavotricuspid isthmus (CTI) dependent and circling the mitral valve annulus] and targeted with appropriate linear ablation lesions with confirmation of bidirectional block.

### Follow-up

Patients were followed at 6–8 weeks, 6 months, and 12 months after the index procedure. Antiarrhythmic drug (AAD) therapy after the ablation procedure was at the discretion of the treating physician. Rhythm monitoring was performed using 12-lead electrocardiograms, trans-telephonic monitoring, periodic 14-day Holter monitors, rhythm strips from wearable devices (e.g. Apple Watch and KardiaMobile), and/or interrogation of insertable cardiac monitors, implantable cardioverter-defibrillators, or permanent pacemakers (when applicable). Recurrence was defined as any documented AF/AT/AFL recurrence lasting ≥30 s after a 90-day blanking period.

### Statistical analysis

We performed propensity score matching to identify separate reference groups for patients with cardiac amyloidosis and cardiac sarcoidosis. First, patients with cardiac amyloidosis were matched to eligible reference patients without infiltrative cardiomyopathy in a 1:4 ratio using nearest-neighbour matching without replacement. Propensity scores were calculated using logistic regression, including age, sex, persistent AF, and heart failure with reduced ejection fraction (HFrEF) as covariates. After matching, reference patients selected for the cardiac amyloidosis cohort were removed from the pool of eligible references before matching patients with cardiac sarcoidosis. For cardiac sarcoidosis, propensity scores were similarly calculated using logistic regression with the same covariates, followed by 1:4 nearest-neighbour matching without replacement. Categorical variables were reported as frequency (percentage) and compared using *χ*^2^ or Fisher’s exact tests, and continuous variables were reported as mean ± standard deviation and compared using Student’s *t*-test. We used logistic regression analysis to assess the association between the presence of cardiac amyloidosis/sarcoidosis and the incidence of non-PV triggers and macro-reentrant flutters. The associations were reported as odds ratios (ORs) with 95% confidence intervals (CIs). Kaplan–Meier curves were used to plot 1-year recurrences among patients with infiltrative cardiomyopathy and their matched reference groups. Patients were censored at time of death or heart transplantation. In addition, we used Cox proportional hazards regression analysis among the matched cohorts to assess the effect of cardiac amyloidosis/sarcoidosis on 1-year risk of arrhythmia recurrence after catheter ablation. Patients who died or underwent repeat AF ablation during the 90-day blanking period as well as those who did not participate in follow-up at our institution were excluded from outcome analyses. The associations were reported as hazard ratios (HRs) with 95% CI. All statistical analyses were performed using SPSS v22 (IBM Corp, Armonk, NY, USA), and a *P*-value of <0.05 was considered statistically significant.

## Results

Among 3524 patients undergoing first-time AF ablation during the study period, 41 patients were diagnosed with infiltrative cardiomyopathies. This included 24 patients with cardiac amyloidosis and 17 patients with cardiac sarcoidosis.

### Cardiac amyloidosis

A total of 24 patients with cardiac amyloidosis were matched to 96 reference patients. Standardized mean difference plot is shown in Supplementary material online, *Figure S1*. Of these, 20 patients had a diagnosis of cardiac transthyretin amyloidosis, and 4 patients had light-chain amyloidosis with cardiac involvement. Patients with cardiac amyloidosis had a mean age of 71.4 ± 6.5 years, and one (4.2%) patient was a woman. Persistent AF was present in 15 (62.5%) patients and HFrEF in 5 (20.8%) patients. A total of 11 patients with cardiac amyloidosis were under treatment with tafamidis at the time of ablation. A detailed description of baseline characteristics in patients with cardiac amyloidosis and their matched reference group is shown in *Table 1*.

**Table 1:** Baseline characteristics of the study sample

	Cardiac amyloidosis			Cardiac sarcoidosis		
	Reference group (*n* = 96)	Cardiac amyloidosis (*n* = 24)	*P*-value	Reference group (*n* = 68)	Cardiac sarcoidosis (*n* = 17)	*P*-value
Age, year	70.8 ± 6.7	71.4 ± 6.5	0.720	59.5 ± 12.1	60.0 ± 11.1	0.878
Female (%)	2 (2.1)	1 (4.2)	0.491	8 (11.8)	2 (11.8)	0.999
Persistent AF (%)	61 (63.5)	15 (62.5)	0.925	36 (52.9)	8 (47.1)	0.664
Hypertension (%)	67 (69.8)	17 (70.8)	0.921	47 (69.1)	10 (58.8)	0.419
Diabetes (%)	20 (20.8)	2 (8.3)	0.239	9 (13.2)	2 (11.8)	0.999
HFrEF (%)	19 (19.8)	5 (20.8)	0.999	28 (41.2)	7 (41.2)	0.999
Coronary artery disease (%)	20 (20.8)	3 (12.5)	0.562	5 (7.4)	3 (17.6)	0.195
Cerebrovascular accident (%)	5 (5.2)	1 (4.2)	0.999	6 (8.8)	2 (11.8)	0.658
Chronic kidney disease (%)	11 (11.5)	4 (16.7)	0.497	6 (8.8)	2 (11.8)	0.658
LVEF, %	53.1 ± 13.1	50.5 ± 12.1	0.388	47.0 ± 15.8	47.4 ± 14.7	0.931
Moderate/severe LA enlargement^a^ (%)	31 (32.3)	13 (54.2)	0.047	22 (32.4)	7 (41.2)	0.493

AF, atrial fibrillation; HFrEF, heart failure with reduced ejection fraction; LA, left atrium; LVEF, left ventricular ejection fraction.

^a^Moderate/severe LA enlargement was defined as LA volume index ≥ 42 mL/m^2^, LA diameter ≥ 4.7 cm in men, or LA diameter ≥ 4.3 cm in women on echocardiography.

Left atrial voltage mapping was performed during sinus/paced rhythm in 91 (75.8%) patients. Among these, left atrial low voltage areas were found more commonly in patients with cardiac amyloidosis compared with their matched reference group [62.5% vs. 17.3%; OR = 7.9 (95% CI: 2.5–25.8), *P* = 0.001]. Following completion of PVI, at least one trigger provocative manoeuvre was performed in 83.3% of patients with cardiac amyloidosis (isoproterenol infusion in 62.5% and atrial burst pacing in 62.5%) and all of the matched reference group (isoproterenol infusion in 90.6% and atrial burst pacing in 74.0%). Patients with cardiac amyloidosis had a significantly higher incidence of non-PV triggers initiating AF or sustained focal AT [29.2% vs. 8.3%; OR = 4.5 (95% CI: 1.4–14.2), *P* = 0.009] compared with the matched reference group (*Table 2*). These non-PV triggers were ablated and included non-PV focal sustained ATs in five (20.8%) patients and non-PV AF triggers in two (8.3%) patients. Macro-reentrant flutters were also more common in patients with cardiac amyloidosis [62.5% vs. 38.5%; OR = 2.7 (95% CI: 1.1–6.7), *P* = 0.038] compared with the matched reference group (*Table 2*). This difference was primarily driven by a significantly higher incidence of LA flutters [37.5% vs. 6.3%; OR = 9.0, (95% CI: 2.8–29.0), *P* < 0.001] rather than CTI-dependent flutters [45.8% vs. 32.3%; OR = 1.8 (95% CI: 0.7–4.4), *P* = 0.217]. In patients with cardiac amyloidosis and LA flutters, a total of 11 macro-reentrant circuits were identified, which included 8 (72.7%) mitral annular/roof dependent flutters and 3 (27.3%) septal flutters. Overall, these procedural findings led to LA posterior wall isolation in 12 (50.0%) patients and mitral annular lines in 9 (37.5%) patients with cardiac amyloidosis, while LA posterior wall isolation was performed in 16 (16.7%) patients and mitral annular lines in 4 (4.2%) patients in the reference group.

**Table 2:** Procedural findings during first-time atrial fibrillation ablation in patients with cardiac amyloidosis compared with matched reference group

	Reference group (*n* = 96)	Cardiac amyloidosis (*n* = 24)	Odds ratio (95% CI)	*P*-value
LA low voltage area (%)	13 (17.3)	10 (62.5)	7.95 (2.45–25.75)	0.001
Non-PV trigger (%)	8 (8.3)	7 (29.2)	4.53 (1.45–14.16)	0.009
AF trigger (%)	4 (4.2)	2 (8.3)	2.09 (0.36–12.15)	0.411
AT trigger (%)	4 (4.2)	5 (20.8)	6.05 (1.49–24.66)	0.012
Macro-reentrant flutter (%)	37 (38.5)	15 (62.5)	2.66 (1.06–6.69)	0.038
CTI-dependent flutter (%)	31 (32.3)	11 (45.8)	1.77 (0.71–4.41)	0.217
LA flutter (%)	6 (6.3)	9 (37.5)	9.00 (2.80–28.96)	<0.001

AF, atrial fibrillation; AT, atrial tachycardia; CI, confidence interval; CTI, cavotricuspid isthmus; LA, left atrium; PV, pulmonary vein.

**Figure 1:**
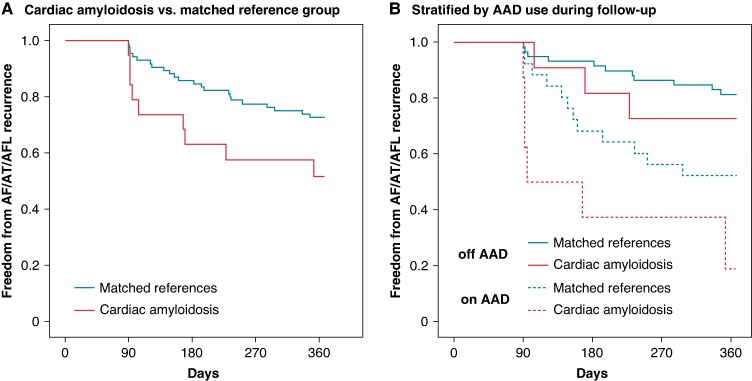
One-year recurrence rate after atrial fibrillation ablation in (*A*) patients with cardiac amyloidosis compared with their respective matched reference groups and (*B*) stratified based on antiarrhythmic drug use during the follow-up. AAD, antiarrhythmic drug; AF, atrial fibrillation; AFL, atrial flutter; AT, atrial tachycardia.

During the follow-up period, 3 patients with cardiac amyloidosis underwent repeat catheter ablation during the blanking period, and 13 patients (including 2 with cardiac amyloidosis) had missing 1-year follow-up. Of the remaining 104 patients who had 1-year follow-up, continuous rhythm monitoring was performed in 31.6% of those with cardiac amyloidosis and 37.6% of the matched reference group. Antiarrhythmic drug therapy continued beyond the 90-day blanking period in 42.1% of patients with cardiac amyloidosis and 30.6% of the matched reference group. At 1-year follow-up, atrial arrhythmia recurrence was significantly higher in patients with cardiac amyloidosis compared with the matched reference group [47.4% vs. 27.1%; HR = 2.2 (95% CI: 1.0–4.7), *P* = 0.049] (*Figure 1A*). In patients off AAD during the follow-up, atrial arrhythmia recurrence was 27.3% in cardiac amyloidosis and 18.6% in the matched reference group; and in those continuing AAD during follow-up, recurrence rate was 75.0% in cardiac amyloidosis and 46.2% in the matched reference group (*Figure 1B*).

### Cardiac sarcoidosis

A total of 17 patients with cardiac sarcoidosis were matched to 68 reference patients. Standardized mean difference plot is shown in Supplementary material online, *Figure S2*. Among these, 14 patients had cardiac sarcoidosis with both cardiac and extracardiac involvement, while 3 patients had isolated cardiac sarcoidosis. Patients with cardiac sarcoidosis had a mean age of 60.0 ± 11.1 years, and two (11.8%) patients were women. Persistent AF was present in eight patients (47.1%) and HFrEF in seven patients (41.2%). A total of seven patients with cardiac sarcoidosis were under treatment with corticosteroids and/or other immunosuppressant agents at the time of ablation. A detailed description of the baseline characteristics in patients with cardiac sarcoidosis and their matched reference group is shown in *Table 1*.

Left atrial voltage mapping was performed during sinus/paced rhythm in 56 (65.9%) patients. Among these patients, left atrial low voltage areas were found more commonly in patients with cardiac sarcoidosis compared with their matched reference group [45.5% vs. 12.2%; OR = 6.0 (95% CI: 1.4–25.8), *P* = 0.017]. Following the completion of PVI, at least one trigger provocative manoeuvre was performed in all patients with cardiac sarcoidosis (isoproterenol infusion in 94.1% and atrial burst pacing in 88.2%) and all of the matched references (isoproterenol infusion in 89.7% and atrial burst pacing in 75.0%). Patients with cardiac sarcoidosis exhibited a numerically higher incidence of non-PV triggers initiating AF or sustained focal AT [17.6% vs. 7.4%; OR = 2.7 (95% CI: 0.6–12.6), *P* = 0.207] compared with the matched reference group; however, this difference did not reach statistical significance (*Table 3*). All non-PV triggers in patients with cardiac sarcoidosis were ablated and included non-PV focal sustained ATs in two (11.8%) patients and non-PV AF triggers in one (5.9%) patient. The incidence of macro-reentrant flutters was similar between patients with cardiac sarcoidosis and the matched reference group [41.2% vs. 32.4%; OR = 1.5 (95% CI: 0.5–4.4), *P* = 0.494] (*Table 3*). Likewise, there were comparable rates of LA flutters [5.9% vs. 4.4%; OR = 1.4, 95% (CI: 0.1–13.9), *P* = 0.799] and CTI-dependent flutters [41.2% vs. 29.4%; OR = 1.7 (95% CI: 0.6–5.0), *P* = 0.354] between the two groups. Overall, these procedural findings led to LA posterior wall isolation in six (35.3%) patients and mitral annular lines in one (5.9%) patient with cardiac sarcoidosis, while LA posterior wall isolation was performed in nine (13.2%) patients and mitral annular lines in three (4.4%) patients in the reference group.

**Table 3:** Procedural findings during first-time atrial fibrillation ablation in patients with cardiac sarcoidosis compared with matched reference group

	Reference group (*n* = 68)	Cardiac sarcoidosis (*n* = 17)	Odds ratio (95% CI)	*P*-value
LA low voltage area (%)	6 (12.2)	6 (54.5)	8.60 (1.99–37.11)	0.004
Non-PV trigger (%)	5 (7.4)	3 (17.6)	2.70 (0.58–12.65)	0.207
AF trigger (%)	2 (2.9)	1 (5.9)	2.06 (0.18–24.19)	0.564
AT trigger (%)	3 (4.4)	2 (11.8)	2.89 (0.44–18.84)	0.268
Macro-reentrant flutter (%)	22 (32.4)	7 (41.2)	1.46 (0.49–4.36)	0.494
CTI-dependent flutter (%)	20 (29.4)	7 (41.2)	1.68 (0.56–5.04)	0.354
LA flutter (%)	3 (4.4)	1 (5.9)	1.35 (0.13–13.90)	0.799

AF, atrial fibrillation; AT, atrial tachycardia; CI, confidence interval; CTI, cavotricuspid isthmus; LA, left atrium; PV, pulmonary vein.

**Figure 2:**
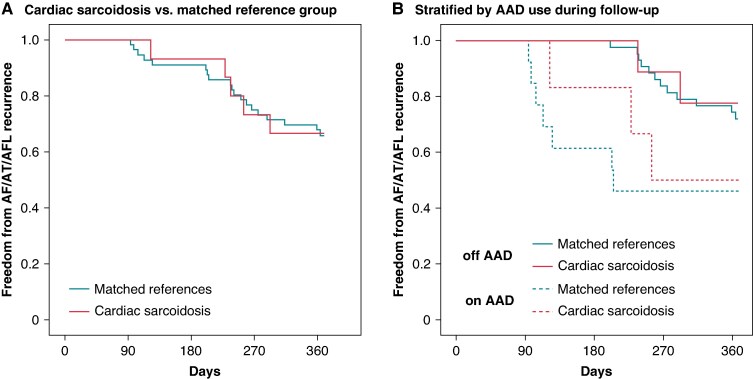
One-year recurrence rate after atrial fibrillation ablation in (*A*) patients with cardiac sarcoidosis compared with their respective matched reference groups and (*B*) stratified based on antiarrhythmic drug use during the follow-up. AAD, antiarrhythmic drug; AF, atrial fibrillation; AFL, atrial flutter; AT, atrial tachycardia.

During the follow-up period, 1 patient from the matched reference group died during the blanking period, 2 patients (including 1 with cardiac sarcoidosis) underwent repeat catheter ablation during the blanking period, and 11 patients (including 1 with cardiac sarcoidosis) had missing 1-year follow-up. Of the remaining 71 patients who had 1-year follow-up, continuous rhythm monitoring was performed in 86.7% of those with cardiac sarcoidosis and 39.3% of the matched reference group. Antiarrhythmic drug therapy continued beyond the 90-day blanking period in 40.0% of patients with cardiac sarcoidosis and 23.2% of the matched reference group. At 1-year follow-up, atrial arrhythmia recurrence was similar across patients with cardiac sarcoidosis [33.3% vs. 33.9%; HR = 0.97 (95% CI: 0.37–2.61), *P* = 0.965] and their matched reference group (*Figure 2A*). In patients off AAD during the follow-up, atrial arrhythmia recurrence was 22.2% in cardiac sarcoidosis and 27.9% in the matched reference group; and in those continuing AAD during follow-up, recurrence rate was 50.0% in cardiac sarcoidosis and 53.8% in the matched reference group (*Figure 2B*).

## Discussion

This study has several key findings. Patients with infiltrative cardiomyopathy undergoing first-time AF ablation have significantly higher rates of left atrial voltage abnormality compared with those without infiltrative cardiomyopathy. These patients also face a two- to four-fold higher risk of focal AF/AT triggers from outside of the PVs and a greater likelihood of having left atrial macro-reentrant flutters. This risk is more pronounced in patients with cardiac amyloidosis than those with cardiac sarcoidosis. At 1-year follow-up, patients with cardiac sarcoidosis demonstrate comparable ablation outcomes with their matched reference group after targeting identified triggers, whereas patients with cardiac amyloidosis still experience higher arrhythmia recurrence rates.

Cardiac amyloidosis and cardiac sarcoidosis, the leading causes of infiltrative cardiomyopathies, have seen a dramatic rise in incidence in recent years.^18–20^ Consequently, a comprehensive understanding of the arrhythmogenic triggers and procedural nuances specific to this patient population is essential for optimizing AF ablation outcomes.^21^ This study is the first to compare procedural findings during first-time AF ablation between patients with and without infiltrative cardiomyopathies. Our findings indicate that the risk of non-PV triggers and left atrial macro-reentrant flutters is substantially higher in patients with cardiac amyloidosis compared with the matched reference group. These findings align with a single-arm study by Kanazawa *et al*.,^22^ who reported non-PV triggers initiating AF or sustained focal AT in 12 (28.6%) out of 42 patients with cardiac amyloidosis undergoing AF ablation. Similarly, Donnellan *et al*.^7^ found that 19 (79%) out of 24 patients with cardiac amyloidosis undergoing AF ablation required additional ablation beyond the PVs. In contrast, data on AF ablation in patients with cardiac sarcoidosis remain significantly limited. One study reported outcomes of cryoballoon AF ablation in four patients with cardiac sarcoidosis and another study reported on five patients undergoing radiofrequency AF ablation, none of which implemented a standard trigger provocation protocol to assess presence of triggers.^10,23^ However, we included the largest cohort of patients with cardiac sarcoidosis undergoing first-time AF ablation to date and found a significantly higher risk of left atrial voltage abnormalities and numerically higher incidence of non-PV triggers in these patients compared with the matched reference group. Nonetheless, the sample size was still insufficient to achieve statistical significance for difference in non-PV triggers, highlighting the need for further confirmatory studies with larger sample size.

The high incidence of left atrial low voltage areas and arrhythmogenic non-PV foci in patients with cardiac sarcoidosis and cardiac amyloidosis points to a shared pathophysiology among patients with infiltrative cardiomyopathies that induces an underlying atrial cardiomyopathy.^24^ First, restrictive left ventricular physiology and altered left atrial wall stress in these conditions can indirectly lead to left atrial enlargement, subsequent scarring, and arrhythmogenesis.^5,25,26^ Second, granulomatous infiltration in the atria in cardiac sarcoidosis and myocardial fibrillar deposition in cardiac amyloidosis can directly create the diseased atrial substrate needed for initiation and perpetuation of arrhythmias.^27,28^ However, despite these pathophysiologic similarities, autopsy studies have shown fibrillar deposition in the atria in over 90% of cardiac amyloidosis cases, while granulomatous involvement of the atria was seen in fewer than 20% of cardiac sarcoidosis cases.^29–32^ These histologic studies suggest a predilection for more extensive atriopathy in cardiac amyloidosis compared with cardiac sarcoidosis.

The exceptionally high risk of non-PV triggers in patients with infiltrative cardiomyopathies emphasizes the need for a special attention and tailored ablation strategy in this unique patient population. The 2024 expert consensus statement on catheter ablation of AF supports the targeted ablation of non-PV triggers to be reasonable, based on several observational studies that highlight their clinical significance.^33^ These studies suggest that patients with identified but untreated non-PV triggers face significantly higher recurrence rates than those without, whereas ablating non-PV triggers can reduce recurrence rates to levels comparable with patients without such triggers.^34–36^ However, patients with cardiac amyloidosis in our study still experienced higher recurrence rates, but those with cardiac sarcoidosis had comparable outcomes with their matched controls. This finding suggests that the durability of targeting these additional triggers and macro-reentrant flutters remains limited in patients with cardiac amyloidosis. This may be explained by the progressive nature of cardiac amyloidosis. The significantly high incidence of left atrial voltage abnormalities, non-PV triggers, and LA flutters in these patients possibly indicates an advanced atrial cardiomyopathy that is prone to generating new arrhythmogenic sources over time. Therefore, an earlier intervention during the disease process may yield more favourable outcomes as higher grades of atrial amyloid deposition have been correlated with a greater risk of atrial tachyarrhythmias.^37^ Future research and collaborative efforts are needed to improve durability of ablation success in patients with cardiac amyloidosis.

### Limitations

This study has some limitations. This was a single-centre observational study, subject to the inherent limitations of observational investigations and possibility of residual confounding. Continuous rhythm monitoring was not performed in all patients during the follow-up period, which may have led to potential under-detection of recurrences in those with opportunistic rhythm monitoring. Information regarding AF burden was not available in our registry and future studies with prospective continuous rhythm monitoring and detailed review would be required to describe AF burden. In addition, the incidence of non-PV triggers or macro-reentrant flutters might be even higher in patients with infiltrative cardiomyopathy, given that fewer patients with cardiac amyloidosis underwent isoproterenol infusion and burst pacing manoeuvres compared with their reference group. All identified triggers were ablated in our study sample, and we were unable to assess the recurrence rate when these triggers were identified but not treated. Although we included the two leading causes of infiltrative cardiomyopathies, we did not include patients with other causes, such as haemochromatosis or Fabry disease. Although this study included the largest cohort of patients with infiltrative cardiomyopathies to date, the sample size and statistical power were still limited for drawing definitive conclusions in this rare population. Therefore, our conclusions should be viewed as hypothesis generating, highlighting the need for future collaborative multi-centre studies with larger sample sizes to confirm our findings.

## Conclusion

Patients with infiltrative cardiomyopathies who undergo first-time AF ablation are at substantially higher risk of having left atrial voltage abnormalities and focal AF/AT triggers from outside of PVs. This risk is particularly more pronounced in patients with cardiac amyloidosis compared with those with cardiac sarcoidosis. Patients with cardiac amyloidosis also exhibit a greater likelihood of having left atrial macro-reentrant flutters during first-time AF ablation. Additionally, the nearly 50% recurrence rate of atrial arrhythmias within the first year of follow-up in patients with cardiac amyloidosis highlights the need for advancements in ablation strategies to achieve more durable outcomes.

## Supplementary Material

euaf100_Supplementary_Data

## Data Availability

The data supporting findings of this study are available from the corresponding author upon reasonable request.
