# Correction to: Catheter ablation of atrial fibrillation in patients with cardiac amyloidosis and sarcoidosis: procedural findings and outcomes

**DOI:** 10.1093/europace/euaf177

**Published:** 2025-08-18

This is a correction to: Alireza Oraii, Balaram Krishna J Hanumanthu (*et al*), Catheter ablation of atrial fibrillation in patients with cardiac amyloidosis and sarcoidosis: procedural findings and outcomes, *EP Europace*, Volume 27, Issue 6, June 2025, euaf100, https://doi.org/10.1093/europace/euaf100

The following changes have been made to the originally published manuscript. The name of the fourth co-author has been emended to read: “Ting-Wei Ernie Liao” instead of: “Ting-Wei Liao”.

