# Supplementary material for: Catheter ablation of atrial fibrillation in patients with cardiac amyloidosis and sarcoidosis: procedural findings and outcomes
Source: Europace. 2025 Jun 20;27(6):euaf100. doi: 10.1093/europace/euaf100 (PMC12204683; doi:10.1093/europace/euaf100)
Supplement: euaf100_Supplementary_Data [file euaf100_supplementary_data.pdf]

Supplemental Material

Supplemental Figure S1. Standardized mean difference plot before and after propensity score matching patients with cardiac amyloidosis

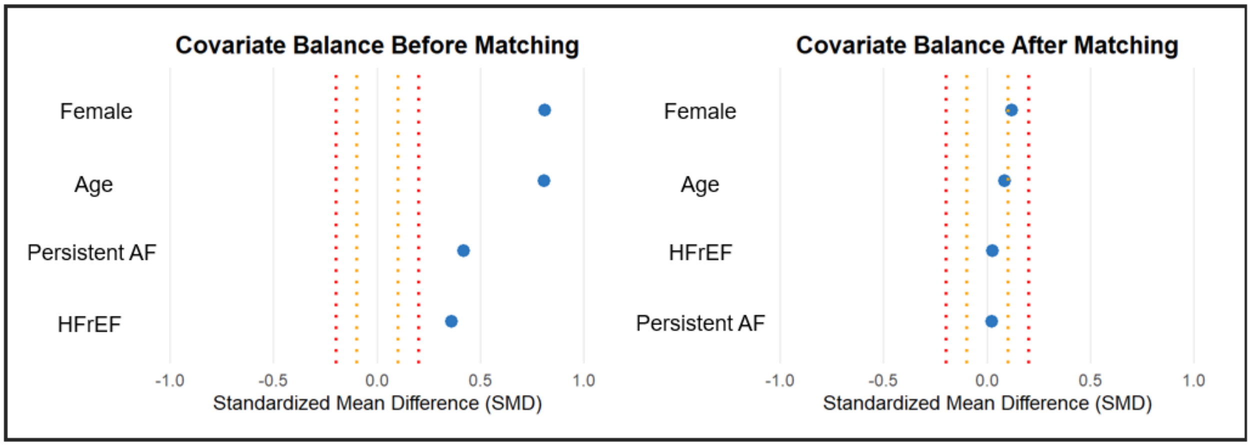

Abbreviations: AF, atrial fibrillation; HFrEF, heart failure with reduced ejection fraction

Supplemental Figure S2. Standardized mean difference plot before and after propensity score matching patients with cardiac sarcoidosis

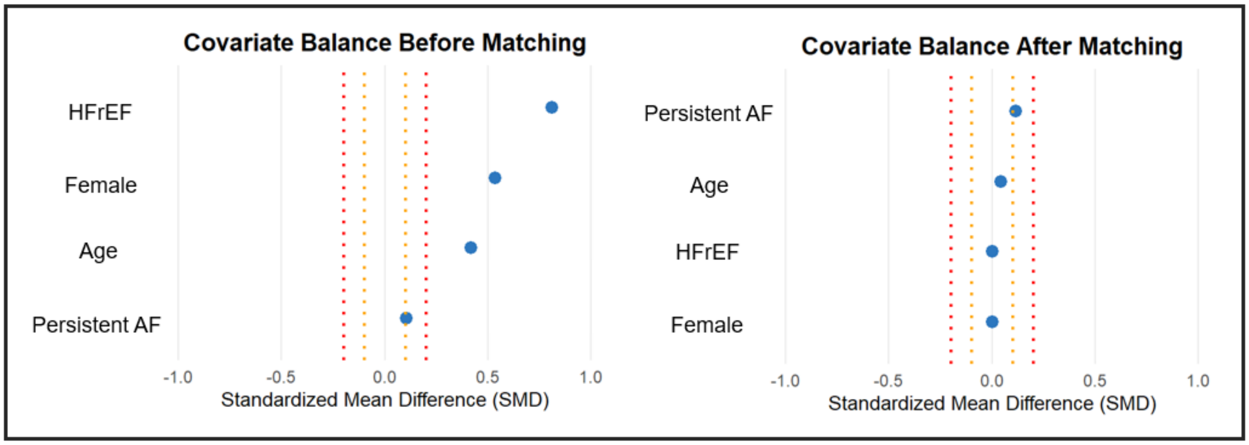

Abbreviations: AF, atrial fibrillation; HFrEF, heart failure with reduced ejection fraction
